# Evidence-Based Strategies to Minimize Unnecessary Primary Cesarean Sections: A Comprehensive Review

**DOI:** 10.7759/cureus.74729

**Published:** 2024-11-29

**Authors:** Nada Y Metwali, Ruqayyah A Ahmed, Jumana Hussain Timraz, Husna Irfan, Samira M Makarfi, Mohammed Y Metwali, Mohammad T Orfali, Jina K Fadl

**Affiliations:** 1 Department of General Medicine and Surgery, Batterjee Medical College for Science and Technology, Jeddah, SAU; 2 Master of Public Health Program, Gulf Medical University, Dubai, ARE; 3 Department of General Surgery, Saudi German Hospital, Dubai, ARE; 4 Department of Obstetrics and Gynaecology, Batterjee Medical College for Science and Technology, Jeddah, SAU

**Keywords:** cesarean section (cs), complications, cs rates, maternal and neonatal outcome, unnecessary

## Abstract

The increase in cesarean section (CS) rates, whether they are classified as unnecessary or elective, has globally raised significant concerns due to the associated risks involving maternal and neonatal outcomes. Although CS can be a lifesaving operation in specific medical cases, its overuse is exposing mothers and neonates to complications like hemorrhage, infections, and long-term consequences such as uterine scarring, infertility, and future pregnancy problems. The contributing factors include maternal preferences for convenience, fear of labor, and financial incentives within the healthcare systems that favor surgical interventions. Defensive medical practices and private healthcare providers further exacerbate this trend. This review discusses the prevalence of CS, highlighting variations between developing and developed regions and the complexity of addressing the rising rates. Moreover, recommendations to reduce unnecessary CS, such as enhancing antenatal education to inform mothers about the risks and benefits associated with different delivery options, promoting supportive care models (midwives), and fostering interdisciplinary cooperation among healthcare providers, will also be addressed. Healthcare systems will gain the ability to reduce the rates of unnecessary Cesarean procedures by directing the main focus on patient education, continuous monitoring, and policy reforms. This will lead to the improvement of both neonatal and maternal health outcomes in addition to lowering the costs of healthcare. In order to provide and ensure evidence-based and safe care for childbirth, a multidisciplinary approach is essential.

## Introduction and background

Women regard childbirth as a significant event in their lives, and an increasing number of them are voluntarily choosing cesarean section (CS) as the mode of delivery. This increase in cesarean section rates presents an issue of concern across many countries [1]. The World Health Organization (WHO) emphasized the concern over the increased rate of cesarean sections in 2015. It declared that a rate of 10% is optimal and a greater rate would indicate no potential benefit in decreasing neonatal and maternal mortality, emphasizing the importance of putting efforts into reducing unnecessary CS [2]. Considering the risks associated with C-sections, it is essential to obtain information on medically approved indications that lead to cesarean delivery rather than vaginal deliveries, including the recommendations for applying appropriate measures to prevent unnecessary cesarean sections [1,3]. Globally, cesarean section rates are increasing due to various reasons, starting from issues related to the healthcare system and healthcare physicians. Hospitals strive to generate revenues and reimburse their finances by allowing operative procedures, which are some of the causes rooted in the system. Financial pressures, private practice, and defensive medicine push physicians into performing more cesarean sections. In addition, some of the causes leading to the rise of the CS trend are due to Maternally Requested Cesarean Section (MRCS) due to the temptation to choose their child’s date of birth, fear of labor pain, or assumption of bodily damage [2]. The contributing factors to this increased rate are complex, and identifying interventions to address them is challenging. A cesarean section is a medical intervention performed to reduce complications that are associated with childbirth [3]. Even though the cesarean section can be identified as a lifesaving procedure for both the child and the mother, there is no evidence indicating the advantages of elective cesarean delivery for women or children who have no medical requirement or indication for the intervention [3]. Unnecessary costly cesarean sections are associated with high maternal and neonatal morbidity and mortality. Like with any other type of surgery, cesarean section is associated with multiple short- and long-term risks and complications that can present later after the current delivery and extend in the following years, affecting the health of either the mother, child, or future pregnancies [3,4]. As with all surgical procedures, it can lead to increased hospital readmissions, surgical complications, and serious risks in subsequent pregnancies, including uterine rupture and preterm birth. Common maternal complications include infections, organ injuries, and hemorrhage, often exacerbated by anesthesia and blood transfusions [4]. Babies delivered via CS are at higher risk for NICU admission and neonatal death, as well as long-term health issues like allergies, low immunity, diabetes, asthma, obesity, and respiratory problems [5,6].

Hence, it is clear that the prevention of unnecessary C-sections can protect both mothers and their babies from the adverse effects and complications of unnecessary surgical procedures [2]. WHO introduced the six blocks that build a healthcare system known as leadership and governance, medical technology, finance, health workforce, information, and delivery of service. These blocks should be put into consideration when forming a healthcare plan [2]. Therefore, the aim of this article is to highlight the key points leading to unnecessary CS rates and to evaluate the effective measures recommended medically in order to achieve a reduction in the rate of unnecessary first cesarean sections.

## Review

Prevalence and epidemiology of cesarean section

Globally, the rates of cesarean sections have seen a dramatic rise over the recent decades, reflecting both changing maternal demographics and medical advancements. The C-section rates in many developed countries are found to hover around 20-30%. They are driven by factors such as maternal age, multiple pregnancies, and a preference for elective procedures [7]. On the contrary, access to safe procedures involving cesarean sections in developing regions still presents a challenge, with rates often below 10%. These lower rates contribute to disparities in neonatal and maternal clinical health outcomes [7]. Since 1990, the average rate of the procedure experienced a rise from 6.7% to 21% until the year 2015 [2]. The WHO experts made an estimate in the year 2010 stating that approximately 6 million cesarean operations, a third of the total, from across the world were regarded as unnecessary procedures [2]. In the Kingdom of Saudi Arabia, the rates of C-sections have been increasing steadily. This increase is in return mirroring global trends, including the recent statistics that suggested estimated rates as high as 25-27% [8]. Cultural attitudes towards childbirth, medical indications such as maternal preference or perceived medical necessity, and the availability of healthcare services are various factors that have played a role in the rise of these rates [8]. In KSA, there is much variation in the epidemiology of cesarean sections across all the regions and hospitals, as it is influenced by different aspects such as healthcare infrastructure, socio-economic factors, and medical practices. In order to monitor and manage these increasing rates, efforts are vital and crucial for ensuring optimal maternal and neonatal health outcomes. Creating a balance between the benefits of C-sections and the potential risks and complications associated with surgical delivery is highly important [7,8].

Identifying clinical scenarios where cesarean section is essential

Vaginal delivery can be challenging in certain clinical scenarios, as it carries specific risks. In contrast, a cesarean section may mitigate these risks and offer a safer alternative in such situations. In fact, it can be beneficial for both mothers and the child. These scenarios are categorized into two main groups [9].

In certain clinical scenarios, vaginal delivery is considered life-threatening for either the mother or the fetus, necessitating a cesarean section (CS). Absolute indications for CS include conditions like cephalopelvic disproportion, where a small maternal pelvis or pelvic deformities prevent vaginal delivery. Infections such as chorioamnionitis require urgent delivery to avoid complications. Life-threatening conditions such as eclampsia and HELLP syndrome also call for CS. Additionally, fetal asphyxia, umbilical cord prolapse, and placenta previa can obstruct safe vaginal delivery, while abnormal fetal positioning or uterine rupture are emergencies that demand immediate cesarean delivery to protect both mother and child [9,10].

Relative indications may also suggest the need for cesarean delivery, including pathological cardiotocography (CTG), which can lead to acute hypoxia or fetal asphyxia; failure to progress in a normal delivery or obstructed labor, arrested or delayed, could potentially harm the fetus or the newborn; previous cesarean sections; most women believe that once you get a cesarean section, having vaginal delivery is no longer an option; however, it’s not always the case, as this option is correct only in case of having more than one cesarean delivery [9,10]. There's always an option for a trial of labor after cesarean (TOLAC)-also known as vagal birth after cesarean (VBAC)-if only one CS was previously performed. Other relative indications include increased maternal age, as it is considered a high-risk pregnancy for a woman over 35 years. While, it’s not an indication for CS, however, older mothers in comparison to younger ones have a higher risk, which might be due to higher chances of hypertension and diabetes; in fact, all morbid conditions increase the risks for complications that can eventually lead to CS [9]. Untreated diabetes, including gestational diabetes, can result in a macrosomic baby, making vaginal delivery difficult to happen. Obesity increases the chances of having type 2 diabetes or developing gestational diabetes, chronic or gestational hypertension, in which all these scenarios favor CS. Fertility treatments often result in higher rates of cesarean delivery, especially due to increased multifetal pregnancy and maternal anxiety about the associated health outcomes [9,10].

In all these indications and scenarios where CS is considered either lifesaving or preferred for better outcomes, in this situation, CS is considered to hold certain advantages; however, this medical advantage is decreasing mortality and morbidity of the mother and the newborn. In contrast, unnecessary first cesarean sections or elective cesarean sections upon maternal request do not offer the same advantages or benefits as they’re viewed as procedures that introduce complications and increase morbidity and mortality that could potentially be avoided. Choosing cesarean delivery when it is not indicated places patients at unnecessary risk [9,10].

Disadvantages and maternal, neonatal long-term complications

With that being said, it can be established that normal vaginal delivery (NVD) is the safest approach for full-term newborns, with no medical necessity hindering NVD or promoting an urgent CS. There are 3 stages of labor leading to vaginal delivery, including the first, second, and third stages of labor. The first stage is further divided into the latent and active stages. The stages involve the start of the contractions and cervical dilations to the delivery of the fetus and placenta, respectively. Throughout these stages, important clinical parameters are monitored for the progression of labor, such as fetal presentation, station, position, and dilation and effacement of the cervix [11]. When monitoring the progression of labor through these clinical parameters, the decision of vaginal delivery can be changed to C-section, if necessary; otherwise, the mode of delivery is usually vaginal [12]. 

As previously mentioned, cesarean delivery carries several disadvantages and long-term complications for both maternal and neonatal health. Other than short-term issues such as a higher risk of wound complications, injury to nearby organs, and side effects from analgesics, there are significant long-term complications. For example, future pregnancies may be at greater risk for placental problems, including placenta accreta spectrum and placenta previa, and even ectopic pregnancy which affects future pregnancies [10,13]. Additionally, many mothers experience chronic headaches and hip pain, affecting over 83% due to needle insertion during spinal anesthesia, potentially leading to spinal fluid leakage [14]. And lastly, a late wound complication, a uterine niche.

The rising rates of cesarean sections have increased concerns about the long-term effects of associated scarring after the procedure. One significant issue that has been increasingly documented is the formation of a uterine niche. A uterine niche is a defect that forms at the site of a cesarean scar, which can lead to various gynecological issues and infertility. Niche is defined as an indentation at least 2 mm deep, often visualized through transvaginal ultrasound [15]. Studies show that 50-60% of women with prior cesarean deliveries may have a visible niche [15,16], which can contribute to symptoms like postmenstrual spotting, dysmenorrhea, and chronic pelvic pain, as well as reduced fertility rates. Notably, its presence has been linked to lower live birth rates after IVF treatments, highlighting its potential role in increasing the current fertility challenges. Innovative surgical procedures, including laparoscopic niche resection, have emerged to address these issues [15].

Here, we come to the other side of these complications, which are neonatal complications and their long-term effects, especially the ones who were born via requested elective cesarean section, presenting with a higher risk of respiratory complications than the ones delivered vaginally. These include respiratory distress syndrome and transient tachypnea of the newborn, which are all short-term complications that can lead to long-term effects like bronchial asthma. A link has been found between cesarean delivery and the occurrence of conditions such as autism, type 1 diabetes, various food allergies, and allergic rhinitis. Although some potential pathophysiological explanations have been proposed, it has yet to be definitively proven [10]. Another complication that can occur after cesarean section is difficulty with breastfeeding. However, there are inconsistencies among studies, with some showing no association and others indicating a negative effect. Delays in mother-child interaction, due to admission to the neonatal unit or spatial separation, may play a role. Nevertheless, this delay appears to have no significant impact on the frequency or duration of breastfeeding after hospital discharge, especially if mothers receive adequate support and advice [10]. However, a vaginal birth increases the mother's chances of nursing her baby successfully in the early postpartum period. In addition, it results in a shorter hospital stay after delivery, promoting a quicker recovery both physically and mentally and strengthening the bond between the mother and the child [17].

Overall, cesarean sections can lead to higher morbidity and mortality rates; for instance, in a study conducted in 2020 on cases seen from 2008-2016, a total of 15,842 fetal deaths were counted out of the total deliveries of 211,741, yielding a fetal mortality rate of about 7%. Total live births over the study period were 211,741, and the total number of maternal deaths was 1500 across participating facilities, yielding an overall maternal mortality ratio of 708 per 100,000 live births [18].

In another study titled ‘Morbidity and Mortality Factors Analysis of Cesarean Section’ based in 2021, the most common complications included bleeding at 50.6%, post-surgical wound infections at 32%, and hysterectomy at 8.6%, with urinary incontinence reported at 7.8% [19].

Therefore, it is typically recommended to promote and support normal vaginal delivery unless cesarean section is indicated for medical reasons; otherwise, cesarean is seen as a disadvantage [13,14]. 

Evidence-based: cesarean section vs. NVD

Elective cesarean sections have been associated with reduced abdominal and perineal pain during and shortly after birth. However, vaginal delivery is associated with shorter hospital stays and a reduced risk of hysterectomy due to postpartum bleeding, which is an advantage that outweighs the reduced pain in CS [20]. Besides, recommendations for elective cesarean sections have evolved, emphasizing that the lowest complication rates are found when these procedures occur during the 39th and 40th weeks of gestation, as earlier deliveries have shown higher rates of respiratory issues in newborns due to lung immaturity and a higher mortality rate [21,22]. While cesarean sections were once thought to protect against urinary incontinence (UI), evidence that vaginal delivery increases UI rates is inconclusive [23], suggesting that this rate is based on other factors, such as prenatal UI [24]. Plus, this protection decreases with subsequent pregnancies, with no evidence of protection with an elective CS versus an emergency-indicated CS. Leading to recommendations against an MRCS for this indication.

Assisted delivery in reducing CS rates

Assisted vaginal delivery is being increasingly recognized as a useful intervention in light of the global rise in CS rates, which is an emerging concern in obstetrics due to its associated risks and long-term health implications. Techniques involved include vacuum extraction and forceps delivery, but both are specifically helpful in situations when labor has arrested in the second stage, maternal exhaustion ensues, or when there are non-reassuring fetal heart patterns. Assisted techniques intervene to facilitate vaginal birth without the need for a CS in conditions where immediate delivery becomes necessary to reduce fetal risks or when immediate interventions might help to avoid a CS, having a successful case outcome, thereby ensuring the safety of both mother and child, especially in high-risk pregnancies or in settings where a quick response is required [25].

The application of assisted vaginal delivery as one of the measures to reduce CS rates needs solid clinical guidelines and training programs for their appropriate and safe use. Assisted delivery is very sensitive regarding technique, and much consideration has been weighed on the proper training of obstetricians to avoid complications. Vacuum and forceps-assisted deliveries have been shown to drastically reduce emergency CS rates when done by skilled practitioners. For example, in well-trained environments, assisted delivery can resolve cases of prolonged second-stage labor with no increasing adverse outcomes for the mother or neonate. Evidence also exists to show that integrating training for assisted delivery into obstetric curricula and ongoing clinical education reduces CS rates by offering clinicians an effective alternative to surgical intervention when complications arise during labor [26,27].

On the one hand, assisted vaginal delivery reduces only individual CS rates, while on the other hand, it acts preventively against recurrent CS and its compounded risks in future pregnancies. A reduction in the primary CS rates is likely to result in notably lower long-term complications in a woman's reproductive life. These women also tend to recover more quickly, have fewer complications in subsequent pregnancies, and report much greater satisfaction with their birth experience. Consequently, promoting assisted deliveries when appropriate according to clinical judgment will influence not only the immediate health of mothers and newborns but also long-term healthy life course trajectories in reproductive health [28].

Assisted delivery in low-resource settings with limited access to surgical facilities also offers an important alternative to avoid unnecessary surgical procedures. High rates of CS impose significant burdens on healthcare resources due to their high economic cost and personnel requirements, especially postoperative care. Assisted vaginal delivery, by reducing the rates of CS, also minimizes the demand for operating room space and postoperative care, thereby increasing the potential sustainability of healthcare services. There is growing evidence from several different healthcare systems that increased availability and appropriate use of assisted delivery techniques can facilitate shorter lengths of hospital stay, reduce healthcare costs, and improve patient throughput, especially in busy maternity units [29].

Financial impact of cesarean section

A study titled ‘Estimating the Financial Impact of Reducing Primary Cesareans’ was conducted at Baystate Medical Center, as part of the American College of Nurse-Midwives’ Reducing Primary Cesareans (RPC) Learning Collaborative, to assess the financial impact of reducing primary cesarean births in nulliparous women with term, singleton, vertex pregnancies (NTSV). It spans a time frame from October 2016 to March 2017. Its goal was to decrease cesarean rates through quality improvement initiatives [30]. 

All women giving birth at the hospital during the specified period were identified, a total of 1747 births. A resource consumption profile was created for different birth types, including vaginal births and cesareans, to assess total hospital costs. The researchers tracked outcomes such as newborn Apgar scores and lengths of stay to ensure that the quality of care remained unaffected during the cesarean reduction efforts [30]. 

Table 1 demonstrates the results of a total of 69 primary cesareans that were prevented, leading to significant cost savings of a total of $413,241, which led to a decrease in the subsequent CS of a total number of 66 CS and a cost of $280,500. The calculated overall cost impact of the reduction of NTSV is $693,741. The study concludes that implementing a systematic approach to reduce NTSV cesareans can lead to substantial hospital cost savings without compromising the quality of care provided to mothers and their newborns. This model can serve as a framework for other hospitals aiming to achieve similar outcomes in cesarean reduction initiatives [30]. 

**Table 1:** Estimate of Total Cost Savings of Reduction in NTSV Cesarean Births in 2016 at Baystate Medical Center: Index and Subsequent Birth [30]

	Savings from Each NTSV Cesarean Prevented $	Number NTSV Cesareans Prevented	Total Savings in NTSV Cesarean Prevented $	Savings from Each Repeat Cesarean Prevented $	Number Repeat Cesarean Prevented	Total Savings in Repeat Cesareans Prevented $	Total Cost Impact of Reducing NTSV Cesareans, 2016 $
Woman	3299	69	227,631	2714	66	179,124	406,755
Newborn	2690	69	185,610	1535	66	101,310	286,920
Total	5989	69	413,241	4250	66	280,500	693,741

Moreover, a systematic review titled "Cesarean sections and for-profit status of hospitals: systematic review and meta-analysis" examined the relationship between hospital profit status (for-profit vs. non-profit) and rates of cesarean sections. The review focused primarily on hospitals in France and the USA, as those countries had the most research available on this topic. Across 17 studies involving over 4.1 million women, the analysis found that CS are significantly more likely to be performed in for-profit hospitals compared to public or private non-profit institutions. The adjusted odds of delivery by CS were 1.41 times higher in for-profit hospitals, with a crude odds ratio of 1.84 [31]. These findings provide strong evidence of an association between hospital profit status and CS rates. This relationship highlights the critical role that financial incentives play in healthcare delivery. The authors suggest these results necessitate a reevaluation of policies to promote appropriate clinical practices and improve patient outcomes, rather than allowing profit motives to unduly influence medical decision-making.

Another article titled "An investigation of Cesarean sections in three Greek hospitals: The impact of financial incentives and convenience”, in Greece-Athens, addresses the significantly rising rates of CS, raising a concern of the underlying factors influencing this trend. This study aimed to explore the various influences on the decision-making for the mode of delivery, focusing on non-medical factors including private health insurance, any form of informal payments in governmental hospitals, physicians' convenience, and the patient's socio-economic status. The study analyzed medical, socioeconomic, and demographic data from births in January 2002 at two public and one private maternity hospital. The results shed light on the complex relationship that exists between medical-clinical judgment and financial incentives in obstetric care and provide a basis for why the rates of cesarean sections are increasing [32]. In private hospitals, having private health insurance was a strong incentive for a CS, as patients with insurance were more likely to opt for a CS compared to those who paid for their deliveries, indicating that financial coverage can influence delivery methods in public hospitals. Additionally, there is a prevalence of informal payments associated with CS deliveries. These payments are often higher for cesareans than for vaginal deliveries, which may incentivize physicians to recommend or perform cesareans to increase their income. The study suggests that these informal payments can be income-dependent, with wealthier women potentially paying more, thus influencing the likelihood of CS [32]. Another reason can be attributed to convenience factors (such as the timing of deliveries), which can motivate physicians to opt for CS, making them outweigh the medical necessity of CS in some cases. Hence, clinicians must make decisions about cesarean sections based solely on medical necessity and the best interests of the patient, rather than being motivated by financial incentives or profit motives. 

Being mindful and cautious when it comes to cesarean deliveries, especially non-indicated primary CS, can not only benefit the patients but also successfully reduce unnecessary healthcare expenditures. This underscores the need for policies and practices that align financial incentives with high-quality, evidence-based care.

Role of physicians in the decision-making process for CS 

As it has been established, almost all mothers spend an awful amount of time trying to decipher what birthing type is better for them, but of course, with the overwhelming anticipation that comes with pregnancy, mothers usually do not have a clear idea of how they would like to welcome their baby to the world, especially with the anxiety that comes with first delivery. Therefore, the overall decision made by the mother can be affected by many factors. Mothers can make clearer, healthier choices if they are well-informed about both delivery options, their pros and cons, and how to optimize recovery time ideally before entering the third trimester. Providing comprehensive, balanced information and support throughout pregnancy can empower mothers to make the best decision for their unique circumstances. As it has already been established, at first, cesarean section was only applied to women who would have a higher mortality rate for delivering a living fetus. Still, nowadays, the cesarean section is used for more than just special cases [33]. 

It is crucial to highlight the impact that healthcare workers have on the decision-making process of selecting an elective CS, as those who favored repeated CS relied on their physicians for this choice. Ensuring that women have access to thorough, balanced, unbiased information and support from healthcare providers can aid in making well-informed decisions that consider both immediate and future delivery choices, as physicians may steer patients towards cesarean sections without thoroughly presenting alternatives or considering patient preferences. This emphasizes the need for improved dialogue and connection between healthcare providers and patients to enhance trust and ensure truly shared decision-making [33].

In a study done by Burns et al., the original article is done to assist physician’s factors on cesarean section decisions. Analyzing data from 33,233 deliveries by 441 physicians across 36 hospitals and one state, the following findings have been found: physician factors contributed more to the explanatory power of the regression. Hi Siri, where are the gig models compared to hospital factors? The most important physician factors influencing CS include prior C-section rates, on Fridays, deliveries between 6 AM and 6 PM, and lower concentration of practice in the hospital. This indicates that the efforts to reduce unnecessary CS should be focused on identifying appropriate clinical indications for CS and disseminating relevant information to physicians [34].

Multidisciplinary approach (MDT)

Reducing the rate of primary cesarean sections emerged as a significant priority within maternal healthcare, given the subsequent risks for both mothers and infants as well as the effect on future pregnancies. A collaborative approach encompassing various healthcare professionals, including obstetricians, nurses, and midwives, is essential. The core role of an obstetrician is to manage labor and delivery effectively, with the primary responsibility of ensuring a safe delivery by assessing and managing labor progress. A nurse’s role before, during, and after a cesarean section is as vital as delivery causes significant anxiety in women, and a nurse’s ability to reassure and communicate with the patient has been shown to improve patient satisfaction and stress reduction. During regional anesthesia placement, the nurse holding that patient’s hand and talking to her through the process can make all the difference in reducing unnecessary primary cesarean section rates [35]. Antenatal education by midwives and doulas provides an opportunity to oppose the normalizing of CS, promote confidence in normal birth, and counter women’s anxieties. These women who had attended classes such as hypnobirthing experienced an increase in their confidence level and felt prepared for normal birth. Which were not routinely available to them [36]. Popular elements of continuous support should be provided by nurses, obstetricians, or midwives during childbirth in the form of emotional support (e.g., continuous presence, reassurance, and affirming words), information about labor progress, and advice regarding coping techniques, comfort measures (e.g., comforting touch, massage, warm baths/showers, encouraging mobility, promoting adequate fluid intake and output), and advocacy (e.g., helping the woman to articulate her wishes to others). The period of support for this intervention varies greatly across studies and contexts. For example, some doula programs may initiate support during pregnancy, provide continuous support during labor and childbirth, and provide support through three months postpartum [37]. 

Comparison of guidelines

Recently updated policies aimed to reduce unnecessary first cesarean sections to address the overuse of this surgical procedure and to overcome the development of higher risks for both mothers and newborns.

In recent times, both national and institutional policies have developed to tackle the high rates of primary cesarean sections. For example, the American College of Obstetricians and Gynecologists (ACOG) and the Society for Maternal-Fetal Medicine (SMFM) have updated their guidelines to promote VBAC and encourage a more conservative use of cesarean deliveries. Their recommendations focus on reducing unnecessary interventions and aids practices that support vaginal births when possible. ACOG's 2021 guidelines emphasize that cesarean delivery should only be performed for well-defined medical indications and stress the importance of patient-centered care in decision-making [38].

The National Institutes of Health (NIH) has aid initiatives with the sole purpose of decreasing cesarean rates through programs that provide evidence-based education to healthcare providers and patients. The NIH's Consensus Development Conference on VBAC underscored the safety of VBAC for many women and encouraged the development of hospital protocols that facilitate this option [39].

Evaluating the influence of these policy changes involves examining both the outcomes of childbirth and the implementation of the new practices. Evidence indicates that when hospitals and obstetricians adhere to updated guidelines, cesarean rates can significantly decline. A fundamental study published in “The Lancet” reviewed global trends in cesarean section rates and pointed out that countries implementing strict policies on elective cesareans observed a slower increase in cesarean rates compared to those without these policies. This suggests that regulatory measures can indeed influence practice patterns on a broader scale. However, there are many challenges in fully assessing the effectiveness of these policies. Variations in how guidelines are implemented across different institutions and regions can affect outcomes [40]. 

Additionally, there is ongoing debate about the balance between reducing cesarean rates and ensuring that women with genuine medical needs receive appropriate care. A commentary by Obstetrics & Gynecology said that while much improvement has occurred due to policy changes, continuous monitoring and adaptation of new challenges will be necessary to assure equity of care [41]. When it comes to the indications about cesarean sections, the guidelines of the National Institute for Health and Care Excellence (NICE), American College of Obstetricians and Gynecologists (ACOG), Royal College of Obstetricians and Gynecologists (RCOG), and the International Federation of Gynecology and Obstetrics (FIGO) have some similarities but also indicate regional differences in practice [42-48]. On the other hand, NICE guidelines are very patient-focused, enabling the woman to choose a Caesarean section, without medical indications for it, only after proper counseling regarding the risks and benefits involved with the procedure. This is in keeping with principles of autonomy in patient decision-making and informed choice [43,44]. In contrast, ACOG advocates the vaginal mode of delivery as being desirable unless there are specific medical conditions that will necessitate intimation for delivery by C-section. They emphasize shared decision-making between the patient and the healthcare provider, with efforts to reduce primary CS, plus their support to VBAC [45,46]. The RCOG guidelines similarly outline detailed indications for both elective and emergency C-sections, including maternal requests after appropriate counseling. They focus on ensuring that the decision is based on medical necessity and patient understanding [47,48]. FIGO's guidelines align with these, advocating for C-sections based on medical need rather than convenience, with a global perspective on reducing unnecessary procedures, particularly in regions with high C-section rates [42]. 

Regarding the preoperative considerations, all guidelines recommend careful patient evaluation and preparation. NICE suggests preoperative investigations include discussion on the most appropriate type of anesthesia together with the timing of the procedure, emphasizing the need for starvation and the use of prophylactic antibiotics to minimize the risk of postoperative infection [43]. ACOG suggests similar recommendations with added emphasis on thromboprophylaxis in those patients who have an increased risk of thromboembolism [44]. Preoperative counseling, prophylactic antibiotics, and thromboprophylaxis preoperatively are also underlined according to the guidelines of RCOG [48]. The recommendations from FIGO were based on the same principles; however, much more attention was thoroughly taken into consideration with regard to adjusting the preoperative practices in resource-limited settings in order for all patients to be ensured of safety and efficacy regardless of their geographical location [42].

The intraoperative techniques recommended by these guidelines are largely consistent, with some variations in emphasis. NICE advises specific surgical techniques to minimize complications, such as using a Pfannenstiel incision and avoiding uterine exteriorization when possible. They also recommend the administration of oxytocin to promote uterine contraction post-delivery [43]. ACOG similarly advocates for the use of a low transverse incision and emphasizes techniques to minimize blood loss, including careful suturing and the use of uterotonics [45]. The RCOG guidelines provide detailed guidance on the surgical approach, including incision type and methods to reduce infection risk and prevent adhesions [48]. FIGO aligns with these recommendations but also considers the challenges faced in low-resource settings, emphasizing the importance of safe surgical practices that are adaptable to varying healthcare environments [42]. Postoperative care is another area where the guidelines converge, although they may differ in their specific recommendations. NICE highlights the importance of pain management, early mobilization, and breastfeeding support, advising that urinary catheters be removed within 24 hours and providing guidance on wound care and discharge planning [43]. ACOG similarly stresses comprehensive postoperative care, including pain management, monitoring for infections, and thromboprophylaxis, along with support for breastfeeding and wound care [45]. The RCOG guidelines emphasize the need for thorough postoperative monitoring, early mobilization, and adequate pain relief, as well as patient education on signs of infection and wound care [48]. FIGO follows these recommendations, with a particular focus on ensuring that postoperative care is tailored to the available resources, aiming to reduce maternal morbidity and mortality globally [42].

NICE encourages offering VBAC to women with a previous C-section, provided they meet specific criteria and are fully informed of the risks and benefits [43]. ACOG is also supportive of VBAC, stating that most women with one previous low transverse C-section are candidates, provided that facilities capable of emergency C-sections are available [44]. The RCOG guidelines recommend VBAC in appropriate candidates and stress the importance of individualized care and counseling [48]. FIGO supports VBAC in selected cases, emphasizing the need for careful patient selection and ensuring that emergency surgical care is available if needed [42]. In summary, while the NICE, ACOG, RCOG, and FIGO guidelines share a common goal of ensuring safe and effective care for both mother and child during and after a C-section, they reflect different regional practices and priorities. NICE is notably patient-centered, with a strong emphasis on informed choice. ACOG focuses on evidence-based practice and minimizing unnecessary C-sections. The RCOG guidelines offer detailed surgical and clinical guidance, while FIGO provides a global perspective, considering the challenges and disparities in C-section access and outcomes across different healthcare settings. Together, these guidelines offer a comprehensive framework for C-section management, adaptable to various clinical and regional contexts [42-48]. Table 2 enlists the guidelines recommended for Cesarean section from four different associations. 

**Table 2:** Guidelines for C-Section [42-45]

	NICE	ACOG	RCOG	FIGO
Indications for CS	Patient choice allowed after counseling	Emphasizes vaginal delivery unless contraindicated	Medical necessity, with patient counseling	Medical reasons prioritized, avoiding unnecessary C-sections
	Clear medical indications	Clear medical indications (e.g., placenta previa)	Indications for both elective and emergency C-sections	Aligns with others, focus on reducing unnecessary C-sections
Preoperative Considerations	Preoperative assessment and discussion	Preoperative assessment, including thromboprophylaxis	Preoperative counseling, antibiotics, thromboprophylaxis	Adapt practices to resource-limited settings
	Use of prophylactic antibiotics	Prophylactic antibiotics	- Similar to NICE and ACOG, focus on comprehensive care	Emphasizes safe and effective care globally
Intraoperative Techniques	Pfannenstiel incision preferred	Low transverse incision preferred	Detailed surgical guidance, including incision type	Safe surgical practices adaptable to varying environments
	Avoid uterine exteriorization when possible	Emphasizes minimizing blood loss	Focus on reducing infection risk and preventing adhesions	Consideration of challenges in low-resource settings
	Use of oxytocin post-delivery	Careful suturing and use of uterotonics		
Postoperative Care	Focus on pain management, early mobilization, breastfeeding	Comprehensive care, including pain management, infection monitoring	Thorough postoperative monitoring, early mobilization	Tailored postoperative care to reduce morbidity and mortality
	Urinary catheter removal within 24 hours	Support for breastfeeding, thromboprophylaxis	Emphasizes patient education on wound care	Aligns with others, but with a global health perspective
Vaginal Birth After Cesarean (VBAC)	Encourages offering VBAC if criteria met	Supports VBAC with facilities for emergency C-section	Recommends VBAC in appropriate candidates	Supports VBAC with careful patient selection
	Requires patient to be fully informed of risks and benefits	Most women with one previous low transverse C-section are candidates	Individualized care and counseling	Ensures emergency surgical care is available if needed

Rates of C-sections in various regions adhering to varied guidelines 

C-section rates vary widely among different countries and regions, with some countries experiencing a significant increase over time [49-52]. For example, in Italy, for which FIGO guidelines have been taken into consideration, the national rate of C-sections increased from 31% in 1998 to 36% in 2002, but with significant regional variation [50]. Similarly, in Pakistan, the rate of C-section increased from 2.7% in 1990-1991 to 15.8% in 2012-2013 [52]. Furthermore, during the decade preceding 1998 in Turkey, the proportion of deliveries by C-section increased from 5.7% to 20.8% [51]. These rates are influenced by factors such as socioeconomic status, education, and urbanization [51-53]. The rates are subject to regional disparities and are influenced by hospital volumes, maternal education, and socioeconomic status, among other factors [50-53]. 

The rates of cesarean section in countries following the ACOG guidelines vary widely. However, Guzman et al. (2015) indicate that some countries in Latin America have C-section rates as high as 50%, suggesting a possible deviation from ACOG guidelines, which emphasize evidence-based practices and patient education to potentially reduce unnecessary C-sections [54]. Laye and Dellinger (2006) provide data from a tertiary care center, showing adherence to ACOG guidelines for the timing of scheduled C-sections but do not specify overall rates [55]. It is interesting to note that Laye and Dellinger add that adherence to ACOG guidelines did not significantly affect neonatal intensive care unit admission rates or neonatal outcomes, which may imply that even adherence to high-intensity guidelines will not correlate with a lesser section rate or an improved outcome [55]. Christopher et al. discuss adherence in the context of prenatal visits rather than in regard to C-section rates [56]. The high rates mentioned for Latin America suggest that there may be a gap between guideline recommendations and practice, but more targeted data would be required to draw definitive conclusions about C-section rates in relation to ACOG guideline adherence [55-56].

The analysis of cesarean section rates across countries following FIGO and ACOG guidelines highlights significant variability in C-section practices. Countries such as Italy, Pakistan, and Turkey, which adhere to FIGO guidelines, have experienced notable increases in C-section rates over recent decades. These rates are influenced by various factors, including socioeconomic conditions, education levels, and urbanization, which contribute to regional disparities. Rates of compliance with ACOG guidelines show that a number of Latin American countries have alarming rates of C-sections, really exceeding recommended practice. However, even with compliance with ACOG recommendations, it is obvious that the recommendations sometimes cannot lead to lower rates of C-sections or better neonatal results, reflecting possible lapses in the translation of evidence-based practices.

On the other hand, there is an absence of adequate information about cesarean section rates with respect to the NICE and RCOG guidelines. This lack of data limits our ability to evaluate and compare the impact of these guidelines on C-section practices. In summary, while existing data provides valuable insights into C-section trends under FIGO and ACOG guidelines, further research is needed to assess the effectiveness of NICE and RCOG guidelines. Addressing this gap will be essential for understanding global C-section practices and improving adherence to evidence-based recommendations.

Discussion

The increasing number of cesarean deliveries poses a complicated issue requiring a detailed review of existing procedures and the adoption of successful strategies to reduce unnecessary surgical births [7]. 

Financial rewards play a role in the increasing rates of CS. Research indicates that in healthcare facilities the availability of health insurance can sway individuals towards choosing a CS due to economic motives trumping clinical reasoning at times. Moreover, unofficial payments linked to deliveries may encourage healthcare professionals to suggest surgical procedures over natural births even when not medically required [31,32]. This pattern not only undermines the standard of healthcare. Also results in higher healthcare expenses, suggesting the immediate requirement for regulations that match financial rewards, with practices supported by evidence [32]. 

Furthermore, the way decisions are made regarding sections often overlooks the need for proper patient education and support. Expectant mothers may find themselves feeling stressed and worried about giving birth, which might make them lean towards choosing a delivery because they believe it to be safer and more convenient. This emphasizes how crucial it is to offer explanations about the pros and cons of both delivery options, enabling women to make well-informed decisions that align with their specific health requirements and personal preferences [33].

In connection with the increasing trend for unnecessary first cesarean sections, several important measures are recommended:

*Enhanced patient education:* focused programs in antenatal education will ultimately help pregnant women gain sufficient knowledge to make appropriate decisions regarding options of delivery. These programs should include discussions on the risks and benefits of both CS and vaginal delivery and the strategies for managing labor pain and anxiety [33,36].

*Supportive care models:* encouragement for continuous support in labor with midwives, doulas, or other skilled health workers will seriously enhance maternal satisfaction and reduce the chance of opting for a cesarean delivery. Many studies have revealed that emotional and physical support during labor will bring about better outcomes with lower rates of unnecessary interventions [35-37].

*Policy reforms:* the health system should aim at reforms that will align the financial incentives with the provision of high-quality, evidence-based care; this would include guidelines on medical needs as indications for CSs and a reduction in the financial pressures to avoid unneeded surgical deliveries [32].

*Interdisciplinary collaboration:* collaboration between obstetricians, midwives, and other health professionals should be fostered in order to provide a coherent approach to childbirth. Shared decision-making models may allow patients to receive balanced information and support so that decisions will result in more appropriate delivery choices [36-37].

*Monitoring and evaluation:* it is very important to continuously monitor the rates and outcomes related to cesarean section delivery to understand how far the measures taken are successful. Regular audits and mechanisms for feedback will establish areas for improvement and make sure practices keep up with current evidence and guidelines [42-48].

Any decrease in rates of cesarean sections that are not absolutely necessary will need to have an intervention at multiple levels targeting patient education, supportive care, and reformation of policies. An environment where there is informed decision-making supported by appropriate financial incentives linked to quality care improves the outcomes for mothers and infants [48].

## Conclusions

In conclusion, the rising rates of unnecessary cesarean procedures present a challenge that has a significant impact on maternal and neonatal health, putting both mothers and neonates at risk while also causing an increase in healthcare costs globally. Although cesarean delivery is a required intervention in specific medical situations and emergencies, its increased use due to non-medical factors such as maternal preferences, financial incentives, and defensive medical practices has led to complications and long-term health consequences. Physicians should follow a balanced way of prioritizing vaginal deliveries and deciding CS for cases with clear medical indications. Addressing these issues in order to decrease the growing rates needs a comprehensive approach, including the improvement of patient education, implementation of supportive care models during labor, fostering interdisciplinary approaches among healthcare professionals, and reforming financial policies that lead to unnecessary surgical interventions. With the implementation of these strategies, healthcare systems worldwide can reduce unnecessary cesarean sections significantly, leading to better neonatal and maternal health outcomes and sustainable health practices.
